# Modeling optical design parameters for fine stimulation in sciatic nerve of optogenetic mice

**DOI:** 10.1038/s41598-021-01353-9

**Published:** 2021-11-19

**Authors:** Nicholas Fritz, Daniel Gulick, Mark Bailly, Jennifer M. Blain Christen

**Affiliations:** 1 Department of Electrical, Computer, and Energy Engineering, Arizona State University, Tempe, 85282 USA; 2 School of Biological and Health Systems Engineering, Arizona State University, Tempe, 85282 USA

**Keywords:** Somatic system, Biomedical engineering, Electrical and electronic engineering, Applied optics, Lasers, LEDs and light sources

## Abstract

Optogenetics presents an alternative method for interfacing with the nervous system over the gold-standard of electrical stimulation. While electrical stimulation requires electrodes to be surgically embedded in tissue for in vivo studies, optical stimulation offers a less-invasive approach that may yield more specific, localized stimulation. The advent of optogenetic laboratory animals—whose motor neurons can be activated when illuminated with blue light—enables research into refining optical stimulation of the mammalian nervous system where subsets of nerve fibers within a nerve may be stimulated without embedding any device directly into the nerve itself. However, optical stimulation has a major drawback in that light is readily scattered and absorbed in tissue thereby limiting the depth with which a single emission source can penetrate. We hypothesize that the use of multiple, focused light emissions deployed around the circumference of a nerve can overcome these light-scattering limitations. To understand the physical parameters necessary to produce pinpointed light stimulation within a single nerve, we employed a simplified Monte Carlo simulation to estimate the size of nerves where this technique may be successful, as well as the necessary optical lens design for emitters to be used during future in vivo studies. By modeling multiple focused beams, we find that only fascicles within a nerve diameter less than 1 mm are fully accessible to focused optical stimulation; a minimum of 4 light sources is required to generate a photon intensity at a point in a nerve over the initial contact along its surface. To elicit the same effect in larger nerves, focusing lenses would require a numerical aperture $> 1$. These simulations inform on the design of instrumentation capable of stimulating disparate motor neurons in mouse sciatic nerve to control hindlimb movement.

## Introduction

The field of bioelectronic medicine instigates therapy in the body by leveraging the neural signals that communicate down to the cellular level^1, 2^. As a form a therapy, stimulating parts of the nervous system has been proven to assist in wound healing and pain reduction^3–5^. Methods for interfacing with the peripheral nervous system (PNS) currently focus on stimulating subsets of fibers within a given nerve bundle^6, 7^. Many implementations of bioelectronic medicine have been limited to the use of cuff electrodes, whose electrical contacts rest only on the nerve surface. To achieve more specific responses, higher resolution stimulation is needed. Neural interfaces for prosthetic devices have addressed this issue by using micro-electrodes or other wire-like structures that embed within a nerve to pinpoint the stimulation^7, 8^. Such multi-electrode arrays have been used to elicit more specific downstream response in muscles by placing electrodes in or against fascicles,(disparate groupings of axons that are the sub-units of a nerve). Although these electrodes and similar devices are used to achieve more discrete stimulus response, electrical stimulation still has its caveats. This kind of intrafasicular stimulation involves precise surgical implantation and suturing of the electrode into the nerve. Not only does this method risk eliciting immune responses that could damage the nerve or incapacitate the electrode, but the insertion into the nerve is blind often resulting in many of the electrode’s contacts being poorly located for recording or stimulus and thus left unused^8–10^.

**Figure 1:**
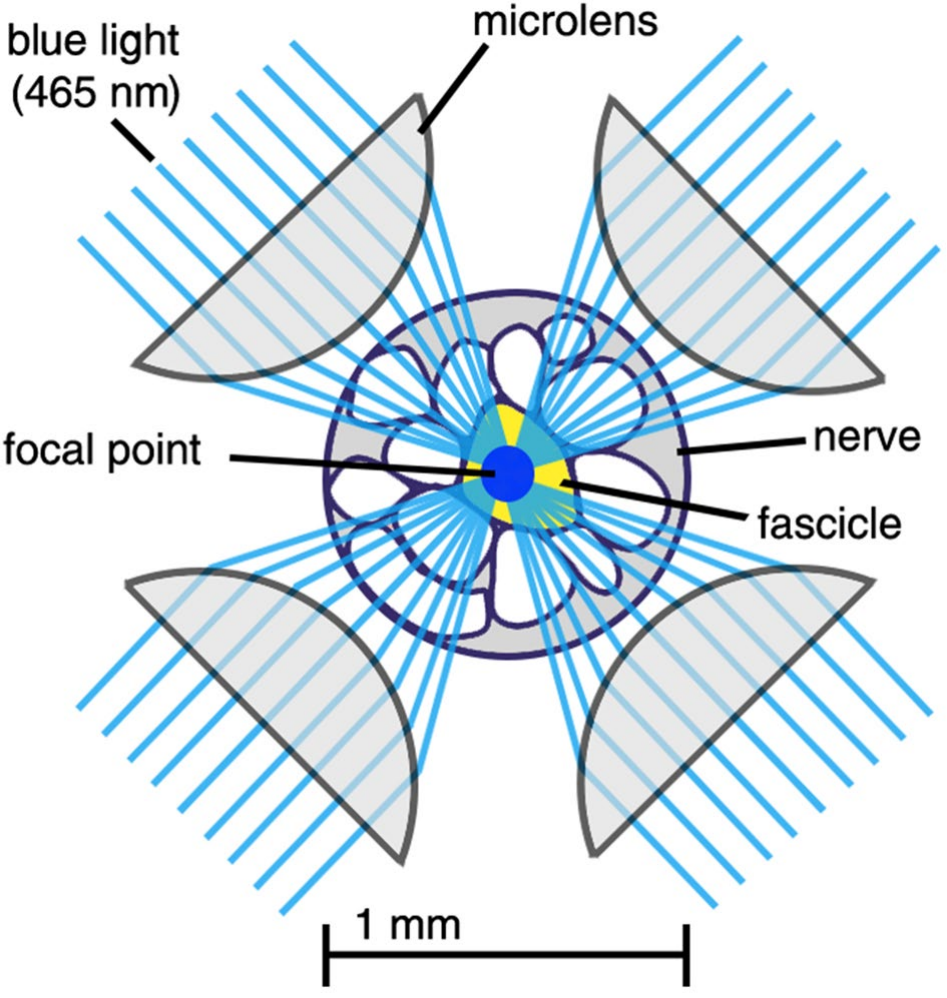
Cross-sectional view of multiple light sources with focused emissions converging on a common focal point; the cholinergic neurons in the fascicle at this location (yellow) are thereby stimulated. It is hypothesized that the photon deposition at the focal point of the multiple emissions will be higher than the photon deposition in any tissue lying in the path of each individual emission. If true, ChR2’s threshold-based stimulus response would allow tuning of emitters so that only the neurons of the fascicle(s) located at the focal point would activate.

Optical stimulation of the nervous system increases specificity of neuron selection while providing little to no damage to the nerve tissue^11–13^. Previous work has ranged from using visible and infrared light pulses to two-photon laser emissions for glutamate uncaging^12–14^. Optogenetics has recently emerged as an alternative method for stimulating the nervous system^15, 16^. By adding genes to the DNA in nerve cells, opsins—a class of protein that changes conformation state upon binding with a photon—can be expressed in the cellular membrane. While opsins vary in function, Channelrhodopsin-2 (ChR2) facilitates a cation transport when presented with blue light ($\lambda =$ 450–490 nm)^17^. Integration of this protein into mammalian neurons enables depolarization of the nerve cell when enough ChR2 proteins in the cell membrane open, thereby instigating an action potential^18–20^. ChR2 is introduced into the mouse genome via the Cre-lox system and results in expression of the protein in cholinergic neurons, including motor neurons of the peripheral nervous system^21^. This ability of optogenetics techniques to reliably confine stimulation to nervous tissues means no other tissue types may be affected or lead to immune response seen with electrical stimulation. To compete with the performance of micro-electrode-interfaces in peripheral nerves an optical stimulator must be able to localize stimulation to small bundles of neurons, known as fascicles, within a single nerve. Optically exciting a single, ChR2-expressing fascicle requires illuminating the target fascicle with enough photons to depolarize the neurons within and without depolarizing other, unwanted neurons between the target and the emitter. To accomplish this, we hypothesize that multiple, focused light emissions can be positioned around the circumference of a peripheral nerve and each emit below the threshold stimulation; the aggregate photon concentration from these focused emissions should exceed the stimulation threshold and depolarize the neurons at the focal point. The physical setup of this hypothesis is represented in Fig. 1.

**Figure 2:**
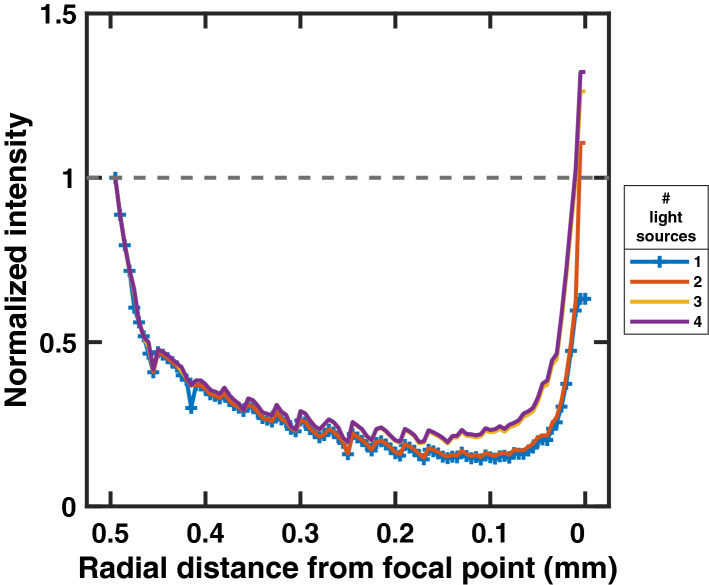
For each 3D simulation environment was summed into a cross-sectional matrix representing a 0.6 mm segment of nerve. The figure shows a simulation of a 1 mm diameter nerve and the change in maximum photon intensity moving from the nerve surface to the center, focal point in a radial manner. Maximum intensity is normalized where the intensity near the nerve surface is 1. The number of light sources simulated ranged from 1–4, showing increased intensity at the focal point with every added light source.

Much of the information that exists regarding light-tissue interaction looks at diffusion and absorption characteristics of different emitters; little exists on optical design for focused emissions passing through tissue. To establish a starting point for fabricating micro-optics (to be used in vivo), we created a simple Monte Carlo (MC) model. The performance of this model was compared against theoretical expected values and also against mcxyz, an MC model written in C to model 3-D light transport through tissue^22^. While many MC packages such as MCML, FullMonte, Dosie, and MOSE can simulate 3D structures and light-absorption through heterogenous mediums, the model presented was built to solely modulate the size, focal power, and number of light sources^23–25^.

## Results

Simulations were performed with collimated, diffuse, and focused light sources. Only focused light sources confirmed the hypothesis that multiple light sources could create a higher photon intensity at the target spot within the nerve than anywhere else in the nerve model. Simulation outputs were analyzed by summation of nerve model cross-sections. As ChR2 is an ion channel expressed in motor neurons of optogenetic mice, its expression in peripheral nerves is limited to the nodes of Ranvier. The internodal distance of the mouse motor neuron is approximately 600 $\upmu$m. In analyzing photon intensity of the simulations, cross-sectional slices of the nerve were summated over this distance; the summated slices were centered about the focal point of the emissions. The resulting matrix was then sectioned into concentric rings to measure maximum photon intensity at the nerve surface and moving inward to the focal point in the center (Fig. 2).

**Figure 3:**
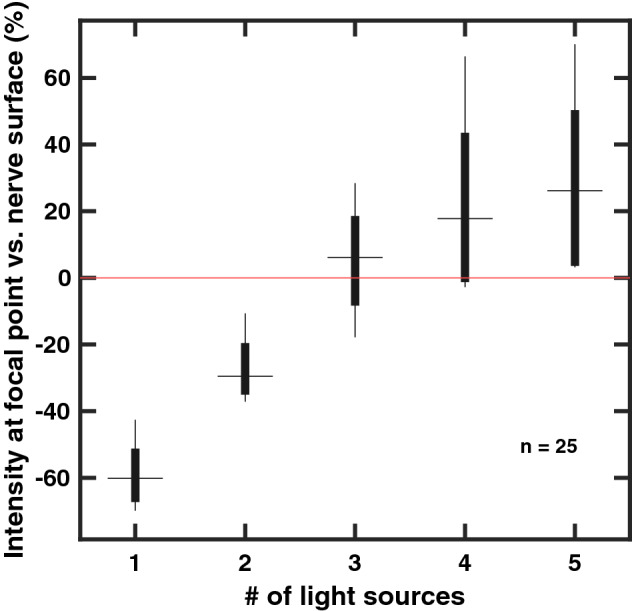
A total of 25 simulations were conducted, with up to 5 light sources simulated. The intensity of the nerve surface for each simulation was normalized to 0 (redline). The use of 4 or more light sources increased the intensity at the target over the nerve surface but was unreliable when using fewer.

**Figure 4:**
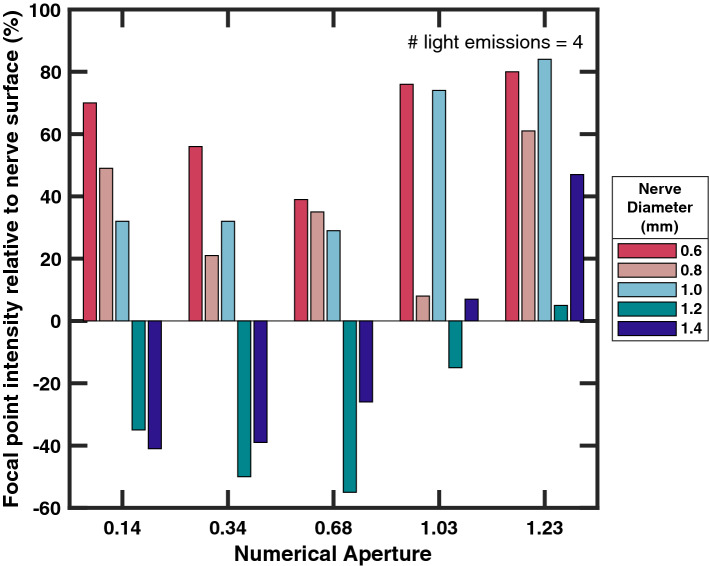
Relative intensity of the focal point to the nerve’s surface as a function of focal power. Focal power is represented by the NA of the lens to provide the angle of focus. The intensity of the nerve surface for each simulation was normalized to 0 with the focal point’s change given as a percentage.

In 25 simulations using fewer than 3 light sources did not increase the photon intensity at the focal point over that of the light’s entry point on the nerve surface (Fig. 3). Successively adding light sources increased photon concentration at the focal point. Simulations of more than 4 light sources showed diminishing returns. The focusing power of an optic, represented as numerical aperture (NA), influenced focal point intensity for larger nerves, as seen in Fig. 4. Increased intensity at the focus would only be possible in nerves larger than 1 mm in diameter by an optic with an NA greater than 1. Using multiple light sources also shows a reduction in NA necessary to stimulate the target, but did not sufficiently bring the NA below 1 for diameters over 1 mm.

**Figure 5:**
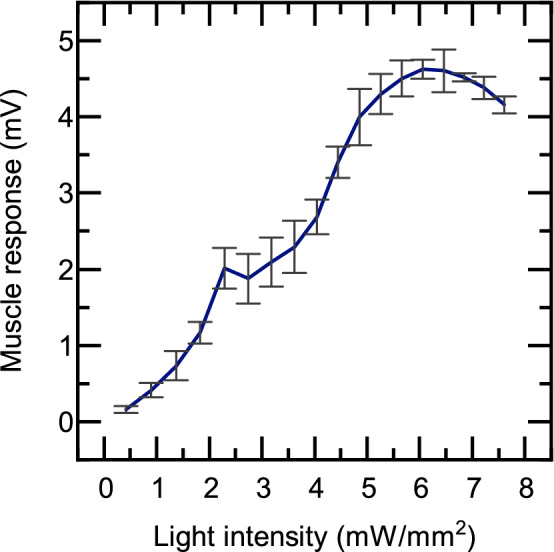
Electrical response from mouse hindlimb muscles during increased intensity of blue light stimulation on the sciatic nerve.

For a system of focused light emissions to successfully stimulate individual fascicles there must be a threshold photon concentration which tissue at the focal point needs to exceed to elicit excitation while surrounding tissue remains sub-threshold. Thus basic in vivo studies were conducted to determine the power threshold for response from ChR2 in peripheral nerves of optogentic mice (ChAT-Cre/Ai32(ChR2-YFP). Increasing light intensity from the LEDs resulted in greater foot flexion and increased electrical response in the muscle. An overall sensitivity curve was generated which indicates a plateau in muscle response when total emissions are above 6 $\hbox {mW/mm}^2$ (Fig. 5). No electrical activity was discernible in the muscle below LED emissions of 0.5 $\hbox {mW/mm}^2$. Both video analysis and EMG recordings showed a hindlimb response that coincided in timing with the light pulse signal. Similarly, no visual response of flexion in the foot could be seen under 1 $\hbox {mW/mm}^2$ of total emission from LEDs. Mouse sciatic nerves ($\hbox {n}=5$) ranged between 0.6 mm to 1 mm in diameter, which were applied to the MC simulations.

**Figure 6:**
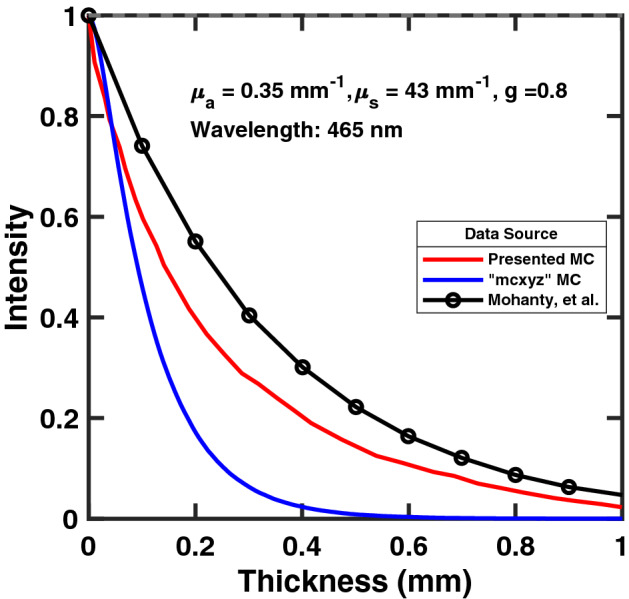
Attenuation curve of blue light (480 nm) in white matter. The blue line shows simulation data of 10k rays, initialized as a collimated beam which is compared against the calculated attenuation from Mohanty et al.^11^.

**Figure 7:**
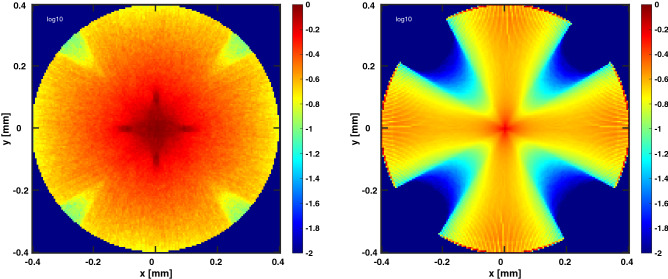
Cross-sectional view of normalized photon energy deposition in a 0.8 mm diameter nerve-analog from mcxyz (left) and the MC model presented (right). Four light sources are used in each simulation (one light source placed at each cardinal point).

The efficacy of the MC simulation was also compared against theoretical attenuation of blue light ($\lambda = 465\,\hbox {nm}$) in white matter, as described by Mohanty, et al.^11^ (Fig. 6). Ten thousand rays were simulated as a collimated, single-light source; slices were taken from the model along the forward direction to measure attenuation of the beam over 1 mm. The MC results show the attenuation seen in the simulation is greater than the calculated attenuation from Mohanty, et al. This slight increase in attenuation from the simulation provides more conservative results than theoretical calculations for absorption and scattering in nerve tissue. Further analysis was performed with mcxyz to determine the attenuation of a single beam initiated with the same homogeneous, white matter properties. The single beam of the model presented attenuated less than that of mcxyz. For further verification a comparison was performed in mcxyz modeling a 0.8 mm diameter nerve, 4 light sources, and using the same attenuation and absorption coefficients as in the model presented. Figure 7 shows a side by side comparison of the results from the two models. Both models indicate that the photon energy deposition is at its highest at the focal point in the center of the tissue.

## Discussion

Simulation parameters were chosen to approximate the anticipated experimental setup for future in vivo studies. The simulated nerve is embedded in air as the simulation results give rough parameterization of the optics necessary for optogenetic stimulation; hardware developed from simulation results will be tested in acute, in vivo procedures using a cuff configuration which will require the nerve to be exposed in open air for implantation. As expected, simulation results indicate that a single light source cannot stimulate a central fascicle within the nerve. A minimum of four light sources were necessary in most simulations to increase photon intensity at the focal point over the intensity at the nerve surface; using more emitters yielded little increase in intensity. Increasing the focal power of emissions benefits larger nerves but still requires a NA over 1. This condition precludes fabrication of regular lenses and to achieve such light gathering capability would require adding elements to the experimental setup, such as lens oil between lenses and the sciatic nerve. Also, increasing focal power of light emissions would increase the spot size of light on the nerve surface; using more light sources may result in overlap of emissions along the nerve surface causing unwanted stimulation of fascicles nearby. For the proposed method of using multiple light sources around the circumference of the nerve to target fascicles near its center, using four light sources coupled with lens optics with NA below 0.68 is recommended.

Performance and function of the model presented is similar to that of mcxyz. Both initialize a forward vector toward a target and use Henyey-Greenstein phase function calculations to determine the distribution of scattering angles based on the tissue’s anisotropy (g). Likewise, after each path length a photon travels, photon energy is attenuated and the energy deposited is stored in voxels; subsequently new scatter parameters are determined and the photons travel in the new direction for a new path length. The result of the focused-beam simulation in mcxyz yields a similar outcome; for a sufficiently small, homogenous nerve model (diameter $< 1.4\,\hbox {mm}$) using focused emissions (lens NA = 0.68), the focal point has the greatest energy deposition.

The results of the MC model only serve as a basis for the fabrication of micro-lenses and emitters to be applied to in vivo studies; the model presented is limited to informing the basic optical design needed for empirical analysis. The simplicity of the MC model does not account for changes in light scattering and absorption properties that would be present in a complex, heterogenous medium. Furthermore, the MC model presented utilized photon packets which decay as a function of distance where more comprehensive models represent individual photons and can track their actual energy throughout a medium. This approach would be required for complex models designed to measure actual photon energy delivered to the layers of tissue within a nerve. While the model uses photon packets which start with a normalized intensity of 1, no units are applied to the output; the aggregate values in the voxels represent a ratio of photon packet energy relative to the initial intensity of the light sources entering at the nerve surface. An estimation of real-world fluence can be generated to apply units to the model results. Assuming photon energy to be $2.2667e^{-3}$ meV ($\lambda$ = 465 nm) per photon, over a period of 1 s, the resulting meV/s per photon can be converted to $3.3617e^{-22}$ mW per photon with the conversion factor $1.6022e^{-19}$. As the simulations use 1.19 million photon packets (rays), and assuming $1e^6$ photons per packet, total energy imparted on the nerve surface would be $4.3217e^{-10}$ mW ($3.3617e^{-22} \times 1.19e^{12}$). The average nerve surface area illuminated during simulations was 0.6249 $\hbox {mm}^2$ and each voxel being $1e^{-10}$ $\hbox {mm}^2$ (10 $\hbox {nm}^2$) results in $6.916e^{-20}$ mW of initialized energy for each illuminated voxel at the nerve surface.

## Methods

### Emission initialization

**Figure 8:**
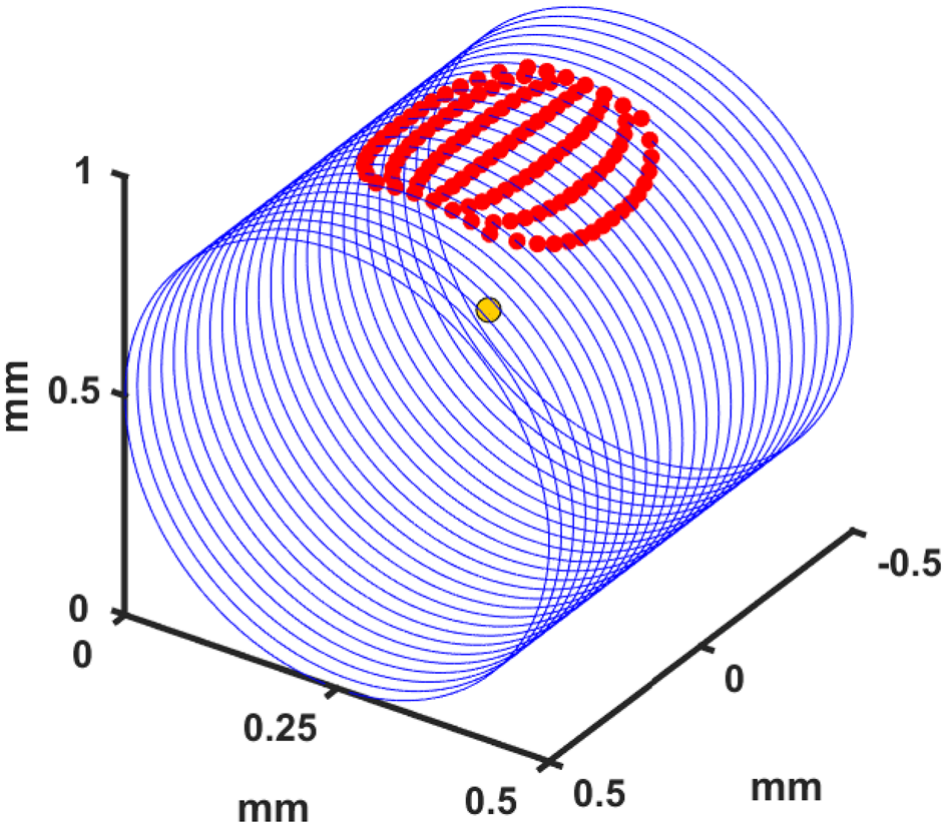
A graphical representation of the 3D environment. Blue rings indicate z-slices of the 1 mm diameter cylindrical nerve. Red points are the photon packet initialization points for a light source with NA of 0.68. The focal point of the overall emission is the center, yellow sphere.

At the start of each simulation, ray initialization points are calculated as a function of focal power. A given NA is converted to mesh of points along the surface of the nerve, which represent the light emission’s contact points, as seen in Fig. 8. Normalized vectors are created for the initial path direction at each point, with each vector pointing at the designated focal point (yellow sphere, Fig. 8). Ten thousand rays are propagated from each surface point (n = 119, 1.19 million total rays per light emission).

### Light scattering and absorption

**Figure 9:**
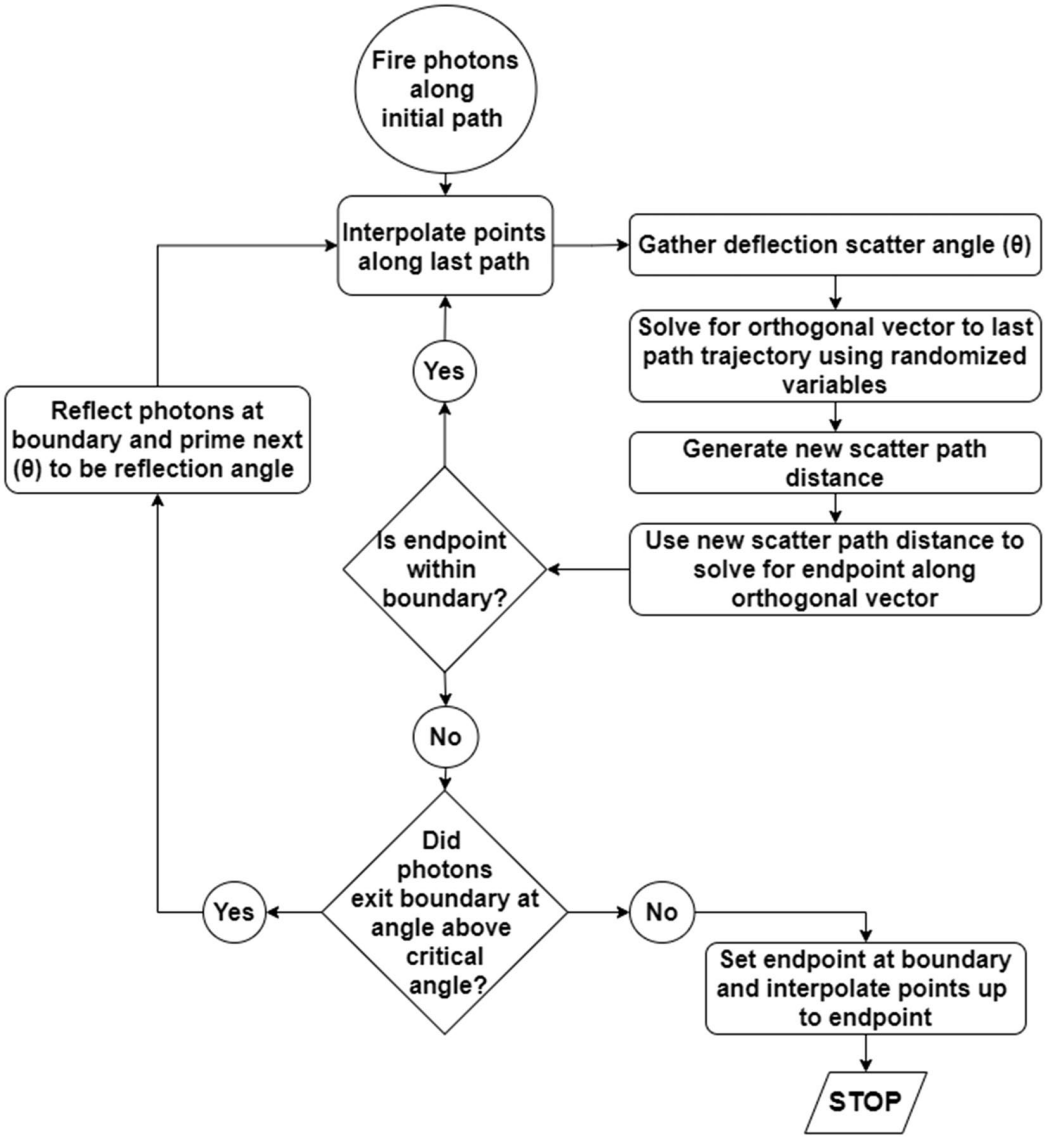
A description of the MC process for simulating light propagation through nerve tissue. Photon rays are initiated in their forward direction and are then scattered and attenuated in accordance with material properties for white matter. Scattering and attenuation are iterated until the photon packet has exited the nerve. Upon exiting the boundary condition, the angle of the packet’s last trajectory was relative to the nerve surface was checked, and if below the critical angle, was reflected and entered the scattering loop again.

Each ray represents a photon packet, which starts with a normalized intensity of 1. In addition to scattering, as a photon packet travels through the 3D model light absorption occurs, attenuating the energy remaining in the photon packet. As latency of ChR2 reaction threshold is 10 ms, it is assumed that there is no time-dependent effect on photon arrival, thus energy deposition can be represented by voxels containing the aggregate intensity of any photon packets that passed through them. As a photon packet passes through voxels, the remaining intensity of the photon packet is added to any existing values in each voxel. The flowchart in Fig. 9 shows the process for light propagation through the cylindrical, homogenous nerve. Each ray is initiated in Cartesian coordinate space with ray trajectories tracked as vectors. Path lengths are determined by an exponential probability distribution where the average scatter distance in tissue is the light scattering coefficient for neural, white matter ($\mu _s = 43$ $\hbox {mm}^{-1}$)^11^. An initial path is randomly selected from the exponential probability distribution and multiplied by the initial vector for the current surface initialization point. Anisotropic scattering is governed by the Heyney–Greenstein (HG) phase function, written as^26^:

1 $$\begin{aligned} P_{HG}(\theta ) = {(1/4\pi )} \times {{(1-g^2)/(1+g^2-2\cdot g \cdot cos(\theta ))^{3/2}}} \end{aligned}$$

Anisotropic scattering (g) in tissue is set to 0.8^11, 27^. Values for $\theta$ range from 0 to $\pi$. The probability of each value of $\theta$ is determined by the Henyey–Greenstein phase function which was used to create a weighted list for the $\theta$ values. Upon scattering, the deflection angle the photon packet scatters from the last vector is ($\theta$) is randomly selected from the weighted list. For each $\theta$ generated at a scatter event, the new direction must be calculated. A randomly-generated vector, orthogonal to the last trajectory’s vector can be used with a new $\theta$ and new path length ($p_n$) to calculate the new direction. Solving the dot product of the last known trajectory, $v_1= {(x_1,y_1,z_1)}$, with an orthogonal vector, $v_2= {(x_2,y_2,z_2)}$, results in the following,

2 $$\begin{aligned} x_1x_2 + y_1y_2 + z_1z_2 = 0 \end{aligned}$$

Since all $v_1$ components are known, two $v_2$ components are randomly selected and assigned a value from a uniform distribution. The remaining $v_2$ component is solved for; the resultant orthogonal vector now describes the direction which the photon will deviate from the previous trajectory. A new path length, $p_n$, is generated and is used as the hypotenuse of the triangle formed by the aforementioned vectors. Using the new $\theta$ the magnitude of each vector can be obtained, ($\Vert \mathbf {v_1} \Vert = p_n$ sin($\theta$), $\Vert \mathbf {v_2} \Vert = p_n$ cos($\theta$)). Adding these vectors to the endpoint of the previous scatter event results in the endpoint of the current scatter event. Before the cycle starts over with the next set of scatter variables, points are plotted from the previous endpoint to the current endpoint, with step size of 10 nm.

The individual points plotted along a ray’s trajectory are used to calculate photon attenuation due to absorption. The decay function used is described by the following;

3 $$\begin{aligned} I = I_0 \times e^{\sqrt{(-3\mu _a (\mu _a+\mu _s(1-g)))}t} \end{aligned}$$

where the absorption coefficient ($\mu _a$ = 0.35 mm) was used to attenuate photon initial energy as a function of total distance traveled in tissue (t)^11^. Rays are initialized with an arbitrary intensity ($\hbox {I}_0$) of 1. After a photon packet exits the boundary condition, t is input as the cumulative summation of the interpolated data points decaying the intensity the further the photon travels. At each scatter event a check is performed if the endpoint exceeds the nerve diameter in the x–y plane. If the endpoint is outside the diameter of the nerve the angle between the photon packets exit vector and the vector normal to the nerve’s surface at the exit point is calculated. This angle is compared against the critical angle, is calculated with Snell’s law ($\theta = sin^{-1}(n_2/n_1)$), where $n_1$ is neural tissue (1.32) and $n_2$ is air (1)^11^. If the exit angle is above the critical angle the photon packet is reflected; a new path length is randomly selected from the exponential probability distribution, and the next randomly-generated scatter angle is overwritten by the reflection angle.

Simulations were performed on the Agave computer cluster at Arizona State University. Individual simulations were parsed across multiples CPU nodes containing two 372 Intel Broadwell CPUs per node. RAM on nodes varied between 128-256 GM and with larger nodes reserved on first come, first serve basis. Overall run-time for a single light source was 59 hours.

### Verification of optogenetic excitation threshold

All experiments with channelrhodopsin expressing mice were were conducted under protocol 16-1483R—approved by the Institutional Animal Care and Use Committee (IACUC)—in addition to approval from the Institutional Biosafety Committee IBC-16-667 112M. All procedures were performed in accordance with IACUC, IBC, and ARRIVE guidelines and regulations. Anesthesia was initiated with intraperitoneal injections of KXA (Ketamine 50 mg/kg, Xylazine 5 mg/kg, Acepromazine 1 mg/kg). Isoflurane anesthesia was administered via an EZ-SA800 Single Animal System (E-Z Anesthesia, Palmer, PA), at 2–$3\%$^28^. Vitals were monitored with a MouseOx Plus system and anesthesia depth was verified by absence of footpad squeeze reflex. Incisions were made caudal to the femur; the proximal end of the incision level with the coxofemoral joint. Dissection through musculature was performed to access the sciatic nerve^28^. A series of three blue ($\lambda = 465\,\hbox {nm}$) light emitting diodes (LEDs) (Cree C503B-BAN-CY0C0461) were placed around the circumference of the sciatic nerve with $30{^\circ }$ rotational translation between them. The LEDs were offset 2 mm from the nerve surface.

We implemented an LED driver circuit to toggle the LEDs at 10 Hz with a $10\%$ duty cycle. LED brightness was increased by step-wise current control from the LED driver and current levels were recorded. After in vivo experimentation, power density of the LEDs was measured with an optical power meter (1830-C, Newport) at current values used during the sciatic nerve stimulation regimen.

Stimulation of the sciatic nerve began with a single LED and was followed by combinations of two and then all three LEDs simultaneously. LEDs were driven at the same current level when used in combination for stimulation. Control light emissions were performed to ensure muscle activation only occurred when the sciatic nerve was presented with blue light. Optical power densities equivalent to that used during the blue light stimulation regimen were presented to the mouse sciatic with UV, green, amber, and IR LEDs with no muscle response detected.
